# Dental caries prevalence in children during temporary protective care according to type of abuse

**DOI:** 10.1186/s12889-024-18833-y

**Published:** 2024-05-18

**Authors:** Yuki Nakamura, Yukiko Nogami, Yoko Iwase, Mio Hozawa, Tetsuya Sotome, Issei Saitoh, Akitsugu Ohuchi, Haruaki Hayasaki

**Affiliations:** 1 Division of Pediatric Dentistry, Niigata University Graduate School of Medical and Dental Sciences, 2-5274 Gakkochodori, Chuo Ward, Niigata, 951-8514 Japan; 2 Department of Oral Health Sciences, Faculty of Health Care Sciences, Takarazuka University of Medical and Health Care, 6-9-38 Nakatsu, Kita Ward, Osaka, 531-0071 Japan; 3 Department of Pediatric Dentistry, Asahi University School of Dentistry, 1851 Hozumi, Mizuho, Gifu, 501-0296 Japan; 4 Department of Dentistry for the Disability and Oral Health, Asahi University School of Dentistry, 1851 Hozumi, Mizuho, Gifu, 501-0296 Japan; 5 Division of Welfare, Niigata University Graduate School of Medical and Dental Sciences, 2-5274 Gakkochodori, Chuo Ward, Niigata, 951-8514 Japan

**Keywords:** Dental caries, Child abuse, Neglect, Children under temporary protective care

## Abstract

**Background:**

This study investigated the correlation between the prevalence of dental caries and the presence and type of abuse.

**Methods:**

Participants were 534 children admitted for care at two child guidance centers (CGCs) in Niigata, Japan. Data pertaining to abuse, including the reason for temporary protective care and the type of abuse, and the oral examination results of the children, were collected. These results were then compared with those of a national survey and analyzed in relation to the presence and type of abuse.

**Results:**

The odds ratio for decayed teeth was 4.1, indicating a higher risk in children admitted to the CGCs. However, no significant association was found between the presence of decayed, filled, or caries-experienced teeth and the presence of abuse. A significant positive association was observed between dental caries and one type of abuse, indicating a greater prevalence of dental caries in cases of neglect. The findings of this study suggest that the type of abuse, rather than its presence, is associated with dental caries.

**Conclusions:**

Our findings suggest that proactive support should be provided to children in problematic nurturing environments, regardless of whether they have been subjected to abuse.

**Supplementary Information:**

The online version contains supplementary material available at 10.1186/s12889-024-18833-y.

## Background

 The percentage of abused children in Japan has been relatively low compared with other developed nations [1–5]; however, the number of reported child abuse cases had been consistently rising since the late 1990s, when the issue was first recognized as a serious social problem. According to a report from the Ministry of Health, Labour and Welfare of Japan (MHLW), the number of abuse cases supported by child guidance centers (CGCs) in the fiscal year of 2020 was 206,301, an increase of 3.6 times over the numbers for the past 10 years [5]. CGCs are public specialized agencies established under a prefecture or an ordinance-designated city to improve the welfare of individual children who for various reasons could be regarded as suffering from one or more attributes of negligence, including abuse, delinquency, and truancy.

The American Academy of Pediatric Dentistry defined dental neglect as “willful failure of parent or guardian to seek and follow through with treatment necessary to ensure a level of oral health essential for adequate function and freedom from pain and infection” [6]. Failure or delay in seeking dental care (dental neglect) causes problems such as pain, suffering, productivity loss (e.g., absences from school), and severe functional and social limitations in affected individuals [7, 8]. Due to the higher prevalence of dental caries among abused children [9–12] and the significant influence of caregivers and the nurturing environment on children’s oral health [13, 14], it is crucial to approach the prevention of child abuse from the perspectives of both dental health and child welfare and take proactive measures against dental neglect. Although neglect of severe dental caries could be seen as a sign of child abuse, some reports show no significant difference in caries prevalence in children granted temporary shelter in CGCs with and without abuse, indicating that caries cannot be directly associated with abuse [15, 16]. Additionally, the relationship between the type of abuse and dental caries has not yet been fully investigated. We found only one study conducted in Toronto, Canada, which reported no difference in caries prevalence between children with different types of maltreatment [11].

Further research is needed to investigate the types of abuse and nurturing environments that may affect the relationship between child abuse and oral health, including dental caries. The present study sought to (1) determine the prevalence of dental caries in children temporarily sheltered at CGCs because of abuse or other reasons, (2) investigate the differences in the prevalence of caries in children with or without abuse, and compare them with the results of the MHLW’s Survey of Dental Diseases, and (3) examine the association between the presence or absence of untreated decayed and caries-experienced teeth (decayed teeth + filled teeth) and the type of abuse. We hypothesized that the type of abuse, rather than the presence of abuse, is associated with the number of untreated teeth leading to dental neglect.

## Methods

This study was designed as a cross-sectional study, and the data collection was performed from January 2015 to July 2019. This study included children admitted for care to two CGCs in Niigata City. One was operated by Niigata Prefecture and the other was operated by Niigata City, an ordinance-designated city. The children for this study were 534 children (308 boys and 226 girls) who had been provided dental examinations in the CGCs from January 2015 to July 2019. Results from the two CGCs were compared with those of the Survey of Dental Diseases performed by the MHLW from October to November 2016. The survey focused on household members aged 1 year and older within 150 areas randomly selected from all regions of the country. The aim of the survey was to understand the dental health status in Japan and obtain foundational data for advancing future dental health measures. The survey data are accessible to the public on the homepage of the MHLW [17], and are recognized as official data for Japan.

The study protocol was reviewed and approved by the Ethics Committee of Niigata University (2015–3036). We obtained informed consent or informed assent from the CGC directors (the temporary custodians of the children) and the children themselves.

A dentist and a dental hygienist from the Pediatric Dentistry Department at Niigata University visited the two CGCs periodically (approximately once a month) to perform dental examinations and interviews about dental health behavior for new children within 2 weeks of them being placed in the CGCs. For children with a history of entering and leaving CGCs multiple times, we used the data from their initial examinations. As for dental health behavior, we interviewed the children to inquire about their toothbrushing habits (frequency) before entering the CGCs. Dental examinations were conducted while the child was seated on a chair, with dental examination instruments such as an LED headlight as appropriate. The dental examination collected information about the degree of caries; the presence and type of dental restorations such as prophylactic fillings, fillings, and prostheses; the presence and degree of plaque buildup; and the presence and severity of gingivitis. Data on abuse of the children were also collected from the two CGCs. These included the reason for temporary protective care, the type of abuse, the primary caregiver, and the abuser. Data were anonymized to prevent the identification of individuals before management and analysis, and sufficient care was taken to protect the confidentiality of personal information.

The numbers of untreated decayed and filled teeth were provided from the oral examinations. The number of individuals with untreated decayed teeth, persons with filled teeth, individuals with caries-experienced teeth (untreated decayed teeth + filled teeth), average number of untreated decayed teeth per person, average number of filled teeth per person, and average number of caries-experienced teeth per person were calculated. All of these data were comparable with corresponding data extracted from the statistical tables of the Survey of Dental Diseases. In both the data from the CGCs and the data extracted from the Survey of Dental Diseases (which focused on individuals under 18 years of age), there were no individuals with permanent tooth loss due to caries. Consequently, we did not address missing teeth in this study. The interviews regarding toothbrushing habits at the CGCs were performed directly with the children, and the analysis included the results for 261 children. Answers that were unclear, such as “don’t remember,” “don’t know,” or “don’t want to answer,” were excluded, along with cases where children could not respond owing to their young age or other reasons. These results were also compared with the corresponding data extracted from the statistical tables of the Survey of Dental Diseases. The Mann–Whitney U test or the Kruskal–Wallis test was used to analyze differences in the number of decayed teeth and caries-experienced teeth, and the χ2 test and residuals analysis were used to analyze the association between the type of abuse and decayed and caries-experienced teeth. Statistical analysis was performed using IBM® SPSS® Statistics version 26 (IBM Japan, Tokyo, Japan) at a significance level of 5%.

## Results

### Characteristics of the study population

Characteristics of the study population in the two CGCs are summarized in Table 1.

**Table 1:** Characteristics of the study population in child guidance centers

Variable			% (*N* = 534)	
Age (years)				
2–5			14.4 (77)	
6–9			25.8 (138)	
10–13			35.4 (189)	
14–18			24.3 (130)	
Sex				
Male			57.7 (308)	
Female			42.3 (226)	
Reason for temporary protective care				
Abuse			60.5 (323)	
Non-abusive reasons			39.5 (211)	
Nursing^a^			19.9 (106)	
Child delinquency			11.6 (62)	
Truancy, confinement			2.4 (13)	
Child disabilities			1.1 (6)	
Other			4.5 (24)	

^a^e.g., Inability to care for the child due to the caregiver’s condition

The proportion of boys was greater than that of girls, with an average age of 10.4 years [standard deviation (SD), 3.85; age range, 2–18 years]. Abuse was the reason for temporary protective care for 60.5% of the children in CGCs. Characteristics of the abused children are shown in Table 2.

**Table 2:** Characteristics of abused children in child guidance centers

Variable		% (*N* = 323)
Age (years)		
2–5		15.8 (51)
6–9		31.6 (102)
10–13		34.4 (111)
14–18		18.3 (59)
Sex		
Male		56.7 (183)
Female		43.3 (140)
Type of abuse		
Physical		54.5 (176)
Neglect		22.3 (72)
Psychological		21.1 (68)
Sexual		2.2 (7)
Main abuser		
Biological mother		48.9 (158)
Biological father		36.8 (119)
Stepfather		5.0 (16)
Brother or sister		1.9 (6)
Stepmother		1.2 (4)
Other		6.1 (20)
Primary caregiver		
Biological mother		64.1 (207)
Biological father		31.3 (101)
Stepmother		0.9 (3)
Stepfather		0.3 (1)
Other		3.4 (11)

### Comparison of the study population with the Survey of Dental diseases

The presence or absence of decayed and filled teeth was compared between the data from the CGCs and the Survey of Dental Diseases. No significant association was found for the presence of filled teeth, but significant positive associations were observed for the presence of decayed teeth and caries-experienced teeth. The odds ratio for decayed teeth was 4.10 (3.7 for 2–6 age range, 4.84 for 7–12 age range, and 1.85 for 13–18 age range) and that for caries-experienced teeth was 2.60 (2.07 for 2–6 age range, 2.06 for 7–12 age range, and 2.03 for 13–18 age range), indicating a higher risk in the CGC children (Table 3).

**Table 3:** Decayed/filled teeth; comparison of data from child guidance centers and the Survey of Dental Diseases

		Frequency (adjusted residual)		χ2	Odds ratio (95%CI)
		Decayed			
		Presence	Absence		
2-6y	CGCs	43 (4.7)	55 (-4.7)	*P* < 0.001	3.70 (2.113–6.482)
	SDD	30 (-4.7)	142 (4.7)		
7-12y	CGCs	136 (7.4)	114 (-7.4)	*P* < 0.001	4.84 (3.134–7.459)
	SDD	38 (-7.4)	154 (7.4)		
13-18y	CGCs	96 (2.3)	90 (-2.3)	*P* < 0.05	1.84 (1.099–3.087)
	SDD	33 (-2.3)	57 (2.3)		
		Frequency (adjusted residual)		χ2	Odds ratio (95%CI)
		Filled			
		Presence	Absence		
2-6y	CGCs	11 (-1.6)	87 (1.6)	n.s.	0.55 (0.265–1.154)
	SDD	32 (1.6)	140 (-1.6)		
7-12y	CGCs	93 (0.0)	157 (0.0)	n.s.	1.01 (0.684–1.490)
	SDD	71 (0.0)	121 (0.0)		
13-18y	CGCs	66 (0.9)	120 (-0.9)	n.s.	1.28 (0.747–2.206)
	SDD	27 (-0.9)	63 (0.9)		
		Frequency (adjusted residual)		χ2	Odds ratio (95%CI)
		Decayed + filled			
		Presence	Absence		
2-6y	CGCs	45 (2.8)	53 (-2.8)	*P* < 0.01	2.07 (1.237–3.470)
	SDD	50 (-2.8)	122 (2.8)		
7-12y	CGCs	154 (3.7)	96 (-3.7)	*P* < 0.001	2.06 (1.407–3.023)
	SDD	84 (-3.7)	108 (3.7)		
13-18y	CGCs	115 (2.7)	71 (-2.7)	*P* < 0.01	2.03 (1.215–3.373)
	SDD	40 (-2.7)	50 (2.7)		

*CGCs* Child guidance centers, *SDD* Survey of Dental Diseases, *CI* Confidence interval, *n.s.* not significant

The mean number of decayed teeth and caries-experienced teeth per patient was significantly higher in the CGCs than in the Survey of Dental Diseases for all age groups, 2–6 years, 7–12 years, and 13–18 years (Table 4).

**Table 4:** Mean number of decayed and filled teeth per person by age group

			N	Mean number of teeth per person (median)					
				Decayed		Filled		Decayed + filled	
Primary teeth	2–6 y	CGC	98	1.6 ± 2.7 ***	(0.0)	0.3 ± 0.9	(0.0)	1.9 ± 3.0 **	(0.0)
		SDD	172	0.7 ± 1.9	(0.0)	0.6 ± 1.7	(0.0)	1.3 ± 2.7	(0.0)
	7–12 y	CGC	250	1.2 ± 2.2 ***	(0.0)	0.7 ± 1.5	(0.0)	2.0 ± 2.8 *	(0.0)
		SDD	192	0.3 ± 0.9	(0.0)	1.0 ± 1.8	(0.0)	1.2 ± 2.1.	(0.0)
Permanent teeth	7–12 y	CGC	250	0.9 ± 1.9 ***	(0.0)	0.2 ± 0.7	(0.0)	1.1 ± 2.1 ***	(0.0)
		SDD	192	0.1 ± 0.4	(0.0)	0.2 ± 0.6	(0.0)	0.3 ± 0.8	(0.0)
	13–18 y	CGC	186	2.1 ± 3.0 ***	(1.0)	0.9 ± 1.5	(0.0)	3.0 ± 3.6 ***	(2.0)
		SDD	82	0.3 ± 0.7	(0.0)	0.7 ± 1.7	(0.0)	1.1 ± 1.9	(0.0)

*SD* Standard deviation, *y* Years, *CGC* Child guidance centers, *SDD* Survey of Dental Diseases

Mean ± SD (median). Results of the Mann–Whitney U test are also shown:

*P < 0.05

**P < 0.01

***P < 0.001

There were no significant gender differences regarding the presence or absence of decayed teeth, filled teeth and caries-experienced teeth in both the CGCs and the Survey of Dental Diseases. The presence of caries-experienced teeth in the CGCs tended to be more frequent among males, although this difference did not reach statistical significance (Table S1).

There were significant differences in terms of toothbrushing frequency between the data from the CGCs and the Survey of Dental Diseases (Table S2). When classified into categories of brushing three or more times a day, twice a day, or once a day or less, a significantly higher number of children in all age groups indicated brushing once a day or less in the CGCs.

### Comparison between abuse and non-abuse cases in CGC

The presence or absence of decayed and filled teeth was compared between children in the CGCs with and without abuse. No significant association was found between the presence of decayed, filled teeth, or caries-experience teeth and the presence of abuse (Table 5).

**Table 5:** Association between the presence or absence of decayed and filled teeth and abuse in CGCs

		Frequency (adjusted residual)		χ2	Odds ratio (95%CI)
		Decayed			
		Presence	Absence		
2-6y	Abuse	27 (-0.9)	39 (0.9)	n.s.	0.69 (0.296–1.618)
	Non-abuse	16 (0.9)	16 (-0.9)		
7-12y	Abuse	88 (-1.4)	84 (1.4)	n.s.	0.68 (0.394–1.163)
	Non-abuse	48 (1.4)	31 (-1.4)		
13-18y	Abuse	49 (1.4)	37 (-1.4)	n.s.	1.49 (0.836–2.667)
	Non-abuse	47 (-1.4)	53 (1.4)		
		Frequency (adjusted residual)		χ2	Odds ratio (95%CI)
		Filled			
		Presence	Absence		
2-6y	Abuse	8 (0.4)	58 (-0.4)	n.s.	1.33 (0.329-5.406)
	Non-abuse	3 (-0.4)	29 (0.4)		
7-12y	Abuse	57 (-1.9)	115 (1.9)	n.s.	0.59 (0.343-1.021)
	Non-abuse	36 (1.9)	43 (-1.9)		
13-18y	Abuse	35 (1.4)	51 (-1.4)	n.s.	1.53 (0.835-2.794)
	Non-abuse	31 (-1.4)	69 (1.4)		
		Frequency (adjusted residual)		χ2	Odds ratio (95%CI)
		Decayed + Filled			
		Presence	Absence		
2-6y	Abuse	29 (-0.6)	37 (0.6)	n.s.	0.78 (0.336-1.827)
	Non-abuse	16 (0.6)	16 (-0.6)		
7-12y	Abuse	100 (-1.5)	72 (1.5)	n.s.	0.64 (0.366-1.129)
	Non-abuse	54 (1.5)	25 (-1.5)		
13-18y	Abuse	58 (1.5)	28 (-1.5)	n.s.	1.56 (0.857-2.848)
	Non-abuse	57 (-1.5)	43 (1.5)		

*CI* Confidence interval

**Fig. 1:**
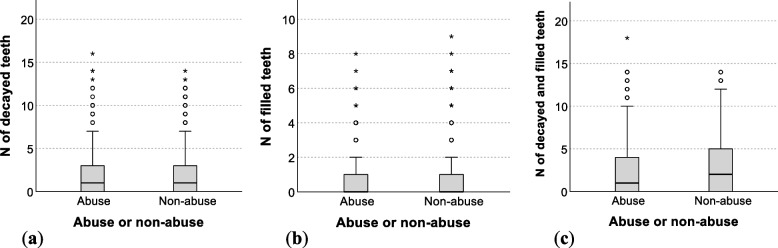
Comparison of the number of decayed and filled teeth between abuse and non-abuse cases (**a**) Number of decayed teeth per person; (**b**) Number of filled teeth per person; (**c**) Number of caries-experienced teeth (decayed teeth + filled teeth) per person The Kruskal–Wallis test was used

 There was no significant difference between children with and without abuse in the number of decayed teeth per person [abuse: mean = 2.0, median = 1.0; non-abuse: mean = 2.2, median = 1.0], the number of filled teeth per person [abuse: mean = 0.7, median = 0.0; non-abuse: mean = 0.9, median = 0.0], and the number of caries-experienced teeth per person [abuse: mean = 2.7, median = 1.0; non-abuse: mean = 3.0, median = 2.0] (Fig. 1). There were outliers with high numbers of decayed, filled, and caries-experienced teeth in both abuse and non-abuse cases.

There were significant gender differences regarding the presence or absence of filled teeth and caries-experienced teeth in abuse cases (Table S3). There were no significant gender differences in non-abuse cases.

There were significant gender differences in the number of filled teeth per person in abuse cases [*P* < 0.05, male: mean = 0.9, median = 0.0; female: mean = 0.6, median = 0.0], the number of caries-experienced teeth per person [*P* < 0.05, male: mean = 2.0, median = 2.0; female: mean = 2.5, median = 1.0].

There was no significant association found when comparing the toothbrushing frequency between CGC children with and without abuse, classified into the categories of three or more times a day, twice a day, and once a day or less.

### Comparison between types of abuse

The presence or absence of decayed and filled teeth was examined between types of abuse, and there was a significant positive association between the presence of decayed in 7–12 age group, caries-experienced teeth and types of abuse, indicating a higher prevalence of decayed teeth and caries-experienced teeth in cases of neglect (Table 6).

**Table:** **Table 6** Association between the presence or absence of decayed and filled teeth and types of abuse

		Frequency (adjusted residual)		χ2
		Decayed		
		Presence	Absence	
2-6y	Physical/sexual	17 (1.3)	18 (-1.3)	n.s.
	Psychological	5 (-1.7)	15 (1.7)	
	Neglect	5 (0.3)	6 (-0.3)	
7-12y	Physical/sexual	46 (-1.8)	55 (1.8)	*P* < 0.001
	Psychological	10 (-2.2)	20 (2.2)	
	Neglect	31 (4.0)	8 (-4.0)	
13-18y	Physical/sexual	28 (0.8)	19 (-0.8)	n.s.
	Psychological	8 (-0.2)	7 (0.2)	
	Neglect	11 (-0.7)	11 (0.7)	
		Frequency (adjusted residual)		χ2
		Filled		
		Presence	Absence	
2-6y	Physical/sexual	4 (0.2)	31 (0.2)	n.s.
	Psychological	4 (1.3)	16 (-1.3)	
	Neglect	0 (-1.3)	11 (1.3)	
7-12y	Physical/sexual	32 (-0.4)	69 (0.4)	n.s.
	Psychological	6 (-1.7)	24 (1.7)	
	Neglect	18 (2.0)	21 (-2.0)	
13-18y	Physical/sexual	19 (0.0)	28 (0.0)	n.s.
	Psychological	7 (0.5)	8 (-0.5)	
	Neglect	8 (-0.5)	14 (0.5)	
		Frequency (adjusted residual)		χ2
		Decayed + Filled		
		Presence	Absence	
2-6y	Physical/sexual	18 (1.3)	17 (-1.3)	n.s.
	Psychological	6 (-1.5)	14 (1.5)	
	Neglect	5 (0.1)	6 (-0.1)	
7-12y	Physical/sexual	55 (-1.2)	46 (1.2)	*P* < 0.001
	Psychological	11 (-2.6)	19 (2.6)	
	Neglect	33 (3.8)	6 (-3.8)	
13-18y	Physical/sexual	33 (0.8)	14 (-0.8)	n.s.
	Psychological	9 (-0.6)	6 (0.6)	
	Neglect	14 (-0.4)	8 (0.4)	

*n.s.* not significant

**Fig. 2:**
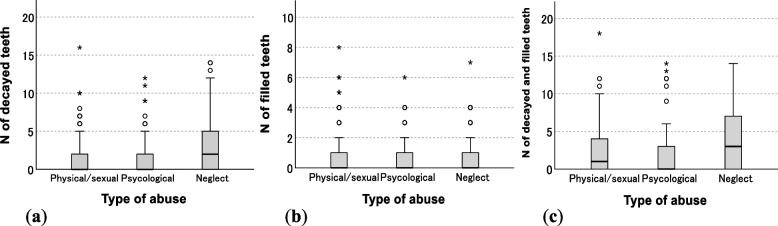
Comparison of the number of decayed and filled teeth between types of abuse (**a**) Number of decayed teeth per person; (**b**) Number of filled teeth per person; (**c**) Number of caries-experienced teeth (decayed teeth + filled teeth) per person The Kruskal–Wallis test was used

 There were significant differences between types of abuse in the number of decayed teeth per person [*P* < 0.01, physical/sexual: mean = 1.5, median = 0.0; psychological: mean = 1.5, median = 0.0; neglect: mean = 3.4, median = 2.0] and the number of caries-experienced teeth per person [*P* < 0.01, physical/sexual: mean = 2.3, median = 1.0; psychological: mean = 2.1, median = 0.0; neglect: mean = 4.1, median = 3.0]. The numbers were higher in neglect (Fig. 2).

There were significant gender differences regarding the presence or absence of decayed teeth, filled teeth and caries-experienced teeth in cases of neglect (Table S4). There were no significant gender differences in cases of physical/sexual and psychological.

There were significant gender differences in the number of filled teeth per person in cases of neglect [*P* < 0.05, male: mean = 1.0, median = 0.0; female: mean = 0.4, median = 0.0], the number of caries-experienced teeth per person [*P* < 0.05, male: mean = 4.8, median = 4.5; female: mean = 3.0, median = 1.5]. The number of decayed teeth in cases of neglect tended to be more common among males, although this difference did not reach statistical significance [*P* = 0.056, male: mean = 3.8, median = 3.0; female: mean = 2.6, median = 0.5].

When examining the toothbrushing frequency based on the type of abuse, there was no significant relationship found between the types of abuse. However, in the 10–14 age group, the number of children who reported toothbrushing less than once a day tended to be higher for neglect, whereas it tended to lower for physical and sexual abuse (Table S5).

## Discussion

The prevalence of dental caries has been reported to be higher in abused and neglected children or children temporarily sheltered at CGCs because of suspected abuse than in the general pediatric population [9–12]. In abused or neglected children the rates of caries-experienced and untreated teeth are high, and the average number of caries-experienced and untreated teeth per capita for these children is considered to be high. An association between caries experience and risk factors indicating neglect has also been suggested [18]. In addition to abuse, other reasons for temporary protective care of children include mental/physical disorders of the caregiver, child delinquency, truancy, confinement, and child disabilities, although some reports show no clear difference in the incidence of dental caries between children in protective care with and without abuse [15, 16]. In this study, the proportions of children with or without decayed teeth, with or without filled teeth, and with or without caries-experienced teeth were compared with the national average reported in the Survey of Dental Diseases. The proportions of children with decayed and caries-experienced teeth were significantly higher among temporarily sheltered children in CGCs, and there was no significant difference in the presence or absence of filled teeth. Additionally, as in previous reports, we found no significant association among temporarily sheltered children in CGCs between the presence of decayed, filled, or caries-experienced teeth and the presence of abuse. The reasons for temporary shelter were divided 60%:40% between child abuse and non-abuse, respectively, in this study. Among the non-abuse reasons, nursing owing to reasons other than child abuse accounted for 20% of the need for care, child delinquency for 10%, and other reasons for 10%. Irrespective of whether the reasons for temporary protective care were related to abuse, the children in the CGCs exhibited a higher prevalence of decayed teeth and caries-experienced teeth compared with the national average, and a lower toothbrushing frequency. This implies that not only abuse but also other inappropriate child-nursing situations may lead to children growing up without adequate care regarding oral hygiene. Multiple caries and dental neglect may reflect the lack of knowledge of oral hygiene of the caregivers, economic deprivation, social isolation, and other inadequacies in the family’s ability to care for the children [18–20]. It is important to recognize that the rate of tooth possession is as high for children with no known background of abuse if the childcare situation is suspected to be inappropriate as it is for children with abuse.

The present study revealed that neglect as one type of abuse is implicated in caries incidence. Children whose abuse was classified as neglect in 7–12 age group had a significantly higher number of decayed teeth. There was no significant difference in the number of treated teeth between neglect and other types of abuse, but in caries-experienced teeth, the total number of untreated and treated teeth was significantly higher in cases of neglect. Neglect results in poor oral health (dental caries, periodontal disease, poor oral hygiene) due to a lack of proper care and, in some cases, the caregivers’ willful failure to seek care even with adequate knowledge [21, 22]. The factor of child abuse, which was separated from neglect after adjustment for potential confounders, showed no significant association with dental caries in a study of Japanese children aged 6–7 years. In contrast, poor involvement and a lack of supervision of a child’s health behaviors were significantly associated with dental caries [23]. In this study, the prevalence of decayed teeth was significantly higher in cases of neglect than that in cases of other types of abuse, although the prevalence of filled teeth was the same. This suggests, in addition to a potentially higher incidence of caries in cases of neglect, the number of filled teeth per person is the same as that for the other types of abuse, indicating that once dental caries occurs, a certain number of affected teeth are likely to be left untreated. However, there were differences in these results by age group. Considering that different age groups may present with different combinations of progressive risks, risk factors, and causes in the development of dental caries, age-related factors must be considered when examining the caries risk factors associated with different abuse types. Physical abuse may result in contusions; burns, or lacerations of the tongue, lips, buccal mucosa, palate (soft and hard), gingiva, alveolar mucosa, or frenum; fractured, displaced, or avulsed teeth; or facial bone and jaw fractures [21, 24]. Sexual abuse may involve the mouth, but visible signs of oral injuries or infections are rare [21]. Our findings also indicate that abuse itself does not increase the risk of dental caries in children, but that the caregiver’s indifference and lack of supervision of children’s health behaviors is most significantly associated with dental caries in children.

We found no gender differences in the presence or absence of caries in both the CGCs as a whole and the Survey of Dental Diseases. However, in cases of abuse where neglect was involved, we did identify gender differences. We noticed that more males had dental caries compared with females, and these males also had a higher number of carious teeth per person. Several reports have suggested that female children and adolescents tend to be more proactive compared with male children and adolescents regarding how often they brush their teeth [25–27]. This observation might be linked to the general tendency for females to be more conscious of their physical well-being and appearance, which in turn makes them more inclined to adopt practices and routines that support good oral health. Without early guidance and support from caregivers, particularly in establishing good oral hygiene practices, such as regular brushing, males might be at a higher risk of developing dental caries compared with females.

Failure to seek or obtain proper dental care may result from factors such as family isolation, lack of finances, lack of transportation, fear of the dental environment as perceived by the child or the parent, and lack of perception of the need for dental care [28]. Even without an abusive background, disadvantaged caregivers have multiple barriers to accessing dental care for their children. In fact, there is an expectation of poor oral health in children in such a socially and economically disadvantaged environment [29]. The Survey of Dental Diseases conducted in 2016 reported that the average number of carious teeth per child in Japan was 2.409 in 6-year-olds and 0.448 in 12-year-olds [17]. Although dental caries are strongly influenced by a nurturing environment, the presence of multiple caries-affected teeth alone cannot be regarded as dental neglect. Harris et al. expressed the view that it would clearly be a vast oversimplification to assume that there is a threshold number of carious teeth, beyond which a diagnosis of dental neglect can be made. Multiple factors have to be considered before diagnosing dental neglect. When dental neglect is suspected, it is important to evaluate the impact of caries on the child, review dental records, assess parental awareness and knowledge, evaluate access to dental care, and consider the child’s willingness to undergo treatment [28, 30].

In this investigation, the primary caregivers were biological mothers for over 50% of the children whose temporary protective care was necessitated by abuse, and these same biological mothers were also identified as the main abusers. Mothers assume a pivotal role in managing the oral hygiene of young children [31, 32] within the domestic setting and accompanying them to dental checkups as prescribed for school-age children. The oral health status of a child could plausibly mirror the caregiving approach and habitat of the primary caregiver. The standing of dentistry in the realm of child abuse has gained significance as a profession entrusted with the prompt identification of child abuse. In some countries, dentists have a mandatory responsibility to follow child protection procedures [30, 33]. In Japan, the amended Child Abuse Prevention Law stipulates that healthcare professionals and other individuals responsible for the well-being of children are required to be cognizant of their ability to identify instances of child abuse and take proactive measures to promptly detect such cases. To detect and aid children and caregivers situated in unsuitable surroundings at the earliest opportunity, it is imperative to scrupulously scrutinize not only the caries status but also the physical well-being, demeanor, and caregiving approach of the child, while taking into consideration the caregiving milieu.

The results of this study should be considered in light of several limitations. Firstly, the category of abuse type in this study was defined as the main reason for temporary protective care. The four types of abuse often overlap and they should not always be considered independently [34]. Secondly, data on abuse were collected from two CGCs, and we did not validate the impact of the parents’ level of education or household income. According to previous research, children’s dental problems and their treatment are impacted by both parental education levels and family functioning [35, 36], and therefore, the relationship between these factors should not be disregarded. Thirdly, the data on the number of carious teeth per person were non-normally distributed, posing a limitation on the analysis. Given that factors related to oral health, such as dental caries, in children whose upbringing is less than ideal are naturally multifaceted, future research models should be designed to accommodate rigorous statistical analyses, such as regression analysis. In this study, since personal, behavioral, nutritional, familial, social, and regional factors that could implicate dental caries activity were not examined, additional analyses of these factors are necessary to enhance the certainty of the study’s conclusions.

## Conclusions

We observed a significantly higher incidence of dental caries in children admitted for temporary care to a CGC when compared with the national standard. However, our analysis did not reveal any significant differences in dental caries prevalence between abused and non-abused children under temporary protective care. When the data were analyzed according to the type of abuse, we found that children classified as neglected had significantly more carious teeth, suggesting that neglect is implicated in the occurrence of dental caries.

Poor or inappropriate childcare environments could be a risk for poor dental health in children. Oral health has emerged as a valuable indicator of child abuse, and dental health practitioners are increasingly called upon to report suspected cases [21]. It is imperative that dental health professionals promptly identify and assist children and caregivers who may be subject to adverse circumstances, such as abuse, at an early stage.

### Supplementary Information

Supplementary Material 1.

## Data Availability

The datasets used and/or analysed during the current study available from the corresponding author on reasonable request.

## Abbreviations

CGC: Child guidance center
MHLW: Ministry of Health, Labour and Welfare of Japan
SDD: Survey of Dental Diseases
CI: Confidence interval
n.s.: Not significant

## References

[1] Child Maltreatment 2020. U.S. Department of Health & Human Services, Administration for Children and Families, Administration on Children, Youth and Families and Children’s Bureau. 2020. https://www.acf.hhs.gov/sites/default/files/documents/cb/cm2020.pdf. Accessed 25 July 2023.

[2] Characteristics of children in need. UK Government. 2022. https://explore-education-statistics.service.gov.uk/find-statistics/characteristics-of-children-in-need. Accessed 25 July 2023.

[3] Child Protection Australia. Australian Institute of Health and Welfare, Australian Government. 2023. https://www.aihw.gov.au/reports/australias-welfare/child-protection. Accessed 25 July 2023.

[4] Ontario Incidence Study of Reported Child Abuse and Neglect. Child Welfare Research Portal, Ministry of Children, Community and Social Services, Government of Ontario. 2020. https://cwrp.ca/sites/default/files/publications/Ontario%20Incidence%20Study%20of%20Reported%20Child%20Abuse%20and%20Neglect%202018.pdf. Accessed 25 July 2023.

[5] Ministry of Health, Labour and Welfare. Handbook of Health and Welfare Statistics 2021. 2022. https://www.mhlw.go.jp/english/database/db-hh/3-2.html. Accessed 25 July 2023.

[6] American Academy of Pediatric Dentistry. Definition of dental neglect. Pediatr Dent. 2018;40:13. 32074833

[7] U.S. Department of Health and Human Services, Public Health Service, Centers for Disease Control and Prevention. Guidelines for school health programs to promote lifelong healthy eating. Centers for Disease Control and Prevention MMWR Recomm Rep. 1996;45:1–41. 8637498

[8] Ayhan H, Suskan E, Yildirim S (1996). The effect of nursing or rampant caries on height, body weight and head circumference. J Clin Pediatr Dent.

[9] Bradbury-Jones C, Isham L, Morris AJ, Taylor J (2021). The neglected relationship between child maltreatment and oral health? An international scoping review of research. Trauma Violence Abuse.

[10] Nogami Y, Iwase Y, Kagoshima A, Saitoh I, Nakajima T, Takahashi H, Nakagawa K, Yoshihara A, Ohuchi A, Asahito T (2017). Dental caries prevalence and treatment level of neglected children at two child guidance centers. Pediatr Dent J.

[11] Valencia-Rojas N, Lawrence HP, Goodman D (2008). Prevalence of early childhood caries in a population of children with history of maltreatment. J Public Health Dent.

[12] Sillevis Smitt H, de Leeuw J, de Vries T (2017). Association between severe dental caries and child abuse and neglect. J Oral Maxillofac Surg.

[13] Dahlan R, Bohlouli B, Saltaji H, Kornerup I, Salami B, Amin M (2022). Immigrant parents’ perceived social support and their children’s oral health behaviors and caries experience. Int J Environ Res Public Health.

[14] Qiu RM, Tao Y, Zhou Y, Zhi QH, Lin HC (2016). The relationship between children’s oral health-related behaviors and their caregiver’s social support. BMC Oral Health.

[15] Kaihara Y, Nizato N, Tachikake M, Mitsuhata C, Kozai K (2016). Overview of community-based oral health support activities at the shelter care facilities and dental caries prevalence of children during temporary custody. Japan J Dent Welf.

[16] Niizato N, Bansyodani A, Otani S, Goto N, lwamoto Y, Yamasaki K, Kozai K (2012). The oral health condition of abused children in temporary shelters in Japan. Japanese J Pediatr Dent.

[17] Survey of Dental Diseases. Ministry of Health Labor and Welfare, Japan. 2016. http://www.mhlw.go.jp/toukei/list/62-17.html. (Japanese only) Accessed 25 July 2023.

[18] Lourenço CB, Saintrain MV, Vieira AP (2013). Child, neglect and oral health. BMC Pediatr.

[19] Peres MA, Macpherson LMD, Weyant RJ, Daly B, Venturelli R, Mathur MR, Listl S, Celeste RK, Guarnizo-Herreño CC, Kearns C (2019). Oral diseases: a global public health challenge. Lancet.

[20] Heima M, Lee W, Milgrom P, Nelson S (2015). Caregiver’s education level and child’s dental caries in African americans: a path analytic study. Caries Res.

[21] Fisher-Owens SA, Lukefahr JL, Tate AR (2017). Oral and dental aspects of child abuse and neglect. Pediatr Dent.

[22] Costacurta M, Benavoli D, Arcudi G, Docimo R (2016). Oral and dental signs of child abuse and neglect. Oral Implantol (Rome).

[23] Matsuyama Y, Isumi A, Doi S, Fujiwara T (2020). Poor parenting behaviours and dental caries experience in 6- to 7-year-old children. Community Dent Oral Epidemiol.

[24] Christian CW, Committee on Child Abuse and Neglect, American Academy of Pediatrics (2015). The evaluation of suspected child physical abuse. Pediatrics.

[25] Mamai-Homata E, Koletsi-Kounari H, Margaritis V (2016). Gender differences in oral health status and behavior of Greek dental students: a meta-analysis of 1981, 2000, and 2010 data. J Int Soc Prev Community Dent.

[26] Salim NA, Alamoush RA, Al-Abdallah MM, Al-Asmar AA, Satterthwaite JD (2021). Relationship between dental caries, oral hygiene and malocclusion among Syrian refugee children and adolescents: a cross-sectional study. BMC Oral Health.

[27] Bhardwaj VK, Sharma KR, Luthra RP, Jhingta P, Sharma D, Justa A (2013). Impact of school-based oral health education program on oral health of 12 and 15 years old school children. J Educ Health Promot.

[28] Bhatia SK, Maguire SA, Chadwick BL, Hunter ML, Harris JC, Tempest V, Mann MK, Kemp AM (2014). Characteristics of child dental neglect: a systematic review. J Dent.

[29] Kelly SE, Binkley CJ, Neace WP, Gale BS (2005). Barriers to care-seeking for children’s oral health among low-income caregivers. Am J Public Health.

[30] Harris JC, Balmer RC, Sidebotham PD (2018). British society of paediatric dentistry: a policy document on dental neglect in children. Int J Paediatr Dent.

[31] Franzman MR, Levy SM, Warren JJ, Broffitt B (2004). Tooth-brushing and dentifrice use among children ages 6 to 60 months. Pediatr Dent.

[32] Finlayson TL, Siefert K, Ismail AI, Sohn W (2007). Maternal self-efficacy and 1-5-year-old children’s brushing habits. Community Dent Oral Epidemiol.

[33] Working together to safeguard children. HM Government. 2006. http://www.familieslink.co.uk/download/june07/working%20together%202006.pdf. Accessed 25 July 2023.

[34] Chiu GR, Lutfey KE, Litman HJ, Link CL, Hall SA, McKinlay JB (2013). Prevalence and overlap of childhood and adult physical, sexual, and emotional abuse: a descriptive analysis of results from the Boston Area Community Health (BACH) survey. Violence Vict.

[35] Pahel BT, Rozier RG, Slade GD (2007). Parental perceptions of children’s oral health: the early childhood oral health impact scale (ECOHIS). Health Qual Life Outcomes.

[36] Arrow P, Klobas E (2015). Evaluation of the early childhood oral health impact scale in an Australian preschool child population. Aust Dent J.

