# Supplementary material for: Dental caries prevalence in children during temporary protective care according to type of abuse
Source: BMC Public Health. 2024 May 18;24:1345. doi: 10.1186/s12889-024-18833-y (PMC11102624; doi:10.1186/s12889-024-18833-y)
Supplement: Supplementary file 1 — Supplementary Material 1. [file 12889_2024_18833_MOESM1_ESM.docx]

**Table S1** Association between the presence or absence of decayed and filled teeth and gender in CGCs

|  | | **Frequency (adjusted residual)** | | **χ2** | **Odds ratio**  **(95%CI)** |
| --- | --- | --- | --- | --- | --- |
|  |  | **Presence** | **Absence** |  |  |
| Decayed | Male | 167 (1.5) | 141 (-1.5) | n.s.  (P = 0.142) | 1.29  (0.917-1.825) |
|  | Female | 108 (-1.5) | 118 (1.5) |  |  |
| Filled | Male | 107 (1.7) | 201 (-1.7) | n.s.  (P = 0.093) | 1.38  (0.948-2.001) |
|  | Female | 63 (-1.7) | 163 (1.7) |  |  |
| Decayed + filled | Male | 192 (1.9) | 116 (-1.9) | n.s.  (P = 0.053) | 1.41  (0.996-2.000) |
|  | Female | 122 (-1.9) | 104 (1.9) |  |  |

CI, confidence interval; n.s., not significant

**Table S2** Toothbrushing frequency; comparison of data from child guidance centers and the Survey of Dental Diseases

|  | | **Frequency (adjusted residual)** | | | **χ2** |
| --- | --- | --- | --- | --- | --- |
|  |  | **Toothbrushing frequency** | | |  |
|  |  | **≥ 3 times / day** | **2 times / day** | **≤ once / day** |  |
| 5-9y | CGC | 4 (-4.8) | 19 (-2.8) | 56 (6.7) | P < 0.001 |
|  | SDD | 36 (4.8) | 46 (2.8) | 22 (-6.7) |  |
| 10-14y | CGC | 9 (-2.6) | 62 (-1.5) | 59 (3.3) | P < 0.001 |
|  | SDD | 18 (2.6) | 57 (1.5) | 24 (-3.3) |  |
| 15-19y | CGC | 3 (-1.9) | 28 (-1.0) | 21 (2.5) | P < 0.05 |
|  | SDD | 15 (1.9) | 57 (1.0) | 19 (-2.5) |  |

CGC, child guidance centers; SDD, Survey of Dental Diseases

**Table S3** Association between the presence or absence of decayed and filled teeth and gender in abuse cases

|  | | **Frequency (adjusted residual)** | | **χ2** | **Odds ratio**  **(95%CI)** |
| --- | --- | --- | --- | --- | --- |
|  |  | **Presence** | **Absence** |  |  |
| Decayed | Male | 101 (1.8) | 82 (-1.8) | n.s.  (P = 0.069) | 1.51  (0.967-2.343) |
|  | Female | 63 (-1.8) | 77 (1.8) |  |  |
| Filled | Male | 66 (2.3) | 117 (-2.3) | P < 0.05 | 1.76  (1.077-2.871) |
|  | Female | 34 (-2.3) | 106 (2.3) |  |  |
| Decayed + filled | Male | 116 (2.3) | 67 (-2.3) | P < 0.05 | 1.68  (1.076-2.632) |
|  | Female | 71 (-2.3) | 69 (2.3) |  |  |

CI, confidence interval; n.s., not significant

**Table S4** Association between the presence or absence of decayed and filled teeth and gender in cases of neglect

|  | | **Frequency (adjusted residual)** | | **χ2** | **Odds ratio**  **(95%CI)** |
| --- | --- | --- | --- | --- | --- |
|  |  | **Presence** | **Absence** |  |  |
| Decayed | Male | 33 (2.2) | 11 (-2.2) | P < 0.05 | 3.00  (1.096-8.214) |
|  | Female | 14 (-2.2) | 14 (2.2) |  |  |
| Filled | Male | 20 (2.1) | 24 (-2.1) | P < 0.05 | 3.06  (1.037-9.000) |
|  | Female | 6 (-2.1) | 22 (2.1) |  |  |
| Decayed + filled | Male | 37 (2.8) | 7 (-2.8) | P < 0.01 | 4.581  (1.529-13.726) |
|  | Female | 15 (2.8) | 13 (2.8) |  |  |

CI, confidence interval; n.s., not significant

**Table S5** Toothbrushing frequency comparison by type of abuse

|  | | **Frequency (adjusted residual)** | | | **χ2** |
| --- | --- | --- | --- | --- | --- |
|  |  | **Toothbrushing frequency** | | |  |
|  |  | **≥ 3 times / day** | **2 times / day** | **≤ once / day** |  |
| 5-9y | Physical/sexual | 2 (1.3) | 8 (-0.3) | 21 (-0.2) | n.s. |
|  | Psychological | 0 (-0.7) | 3 (0.2) | 7 (0.1) |  |
|  | Neglect | 0 (-0.8) | 4 (0.1) | 10 (0.2) |  |
| 10-14y | Physical/sexual | 4 (1.0) | 22 (1.5) | 9 (-2.2) | n.s.  (P = 0.056) |
|  | Psychological | 1 (-0.3) | 10 (0.8) | 5 (-0.7) |  |
|  | Neglect | 1 (-0.8) | 8 (-2.4) | 15 (2.9) |  |
| 15-19y | Physical/sexual | 0 (-1.6) | 9 (0.7) | 5 (0.1) | n.s. |
|  | Psychological | 1 (0.6) | 5 (0.3) | 2 (-0.7) |  |
|  | Neglect | 1 (1.4) | 1 (-1.4) | 2 (0.7) |  |

n.s., not significant
